# ID 108 - Rosuvastatina para o Tratamento de Indivíduos com Alto a Muito Alto Risco Cardiovascular: revisão sistemática e metaanálise

**DOI:** 10.5327/2237-9622.2025.v34s1.108

**Published:** 2025-11-25

**Authors:** Isabela Pina Meza, Tassiane Cristine Santos de Paula, Mariana Millan Fachi, Haliton Alves Oliveira, Rosa Camila Lucchetta, Layssa Andrade Oliveira

**Keywords:** rosuvastatina, estatinas, doenças cardiovasculares, fatores de risco de doenças cardíacas, ensaios clínicos controlados aleatórios

## Abstract

**Introdução:**

As estatinas são amplamente utilizadas e comprovadamente eficazes na redução dos níveis de colesterol LDL e no aumento do colesterol HDL, desempenhando papel essencial na prevenção de doenças cardiovasculares. No Sistema Único de Saúde (SUS), as estatinas disponíveis para o tratamento de dislipidemias e prevenção cardiovascular incluem atorvastatina, sinvastatina e pravastatina. A introdução de novas opções terapêuticas, como a rosuvastatina, pode oferecer benefícios adicionais para pacientes com alto e muito alto risco cardiovascular. Nesse contexto, a análise da rosuvastatina é relevante para otimizar os tratamentos disponíveis e melhorar os desfechos clínicos desses pacientes. Assim, este estudo tem como objetivo avaliar a eficácia e a segurança da rosuvastatina no tratamento de pacientes com alto a muito alto risco cardiovascular, em prevenção primária e secundária, comparando seu desempenho terapêutico com as estatinas incorporadas no SUS.

**Método:**

Foi realizada uma revisão sistemática buscando por ensaios clínicos randomizados (ERC) no PubMed e Embase. Os desfechos primários foram os eventos adversos cardiovasculares maiores (MACE, um desfecho composto que, na maioria dos estudos, englobou morte por doença cardiovascular, acidente vascular cerebral e infarto do miocárdio). Os desfechos secundários incluíram alterações no perfil lipídico (LDL-c, HDL-c e não HDL-c) e a ocorrência de eventos adversos (EA). A avaliação do risco de viés foi conduzida utilizando a ferramenta ROB 2.0, e a qualidade das evidências foi graduada conforme o sistema GRADE. Metanálises diretas foram realizadas para o desfecho de alteração do perfil lipídico.

**Resultados:**

Foram incluídos 22 ECR. Para o desfecho MACE, não houve diferença significativa entre o tratamento de alta intensidade com rosuvastatina e atorvastatina (HR: 1,06, IC 95% 0,86 a 1,30), assim como para os componentes MACE quando avaliados separadamente. Em relação ao desfecho de alteração de perfil lipídico, a rosuvastatina resultou em redução média percentual de -0,04 (IC 95%: -0,06 a -0,02) mg/dl nos níveis de LDL-c, de -0,03 (IC 95%: -0,04 a -0,01) mg/dl nos níveis de não HDL, além de um aumento de 0,02 (IC 95%: 0,01 a 0,04) mg/dl nos níveis de HDL. Esses efeitos foram consistentes tanto na prevenção primária quanto na secundária. Embora o tratamento com rosuvastatina tenha apresentado maior número de eventos adversos gerais (OR: 1,75; IC95%: 1,17-2,61) em comparação com a atorvastatina, não houve diferença quanto aos eventos adversos graves. A qualidade da evidência foi considerada moderada para MACE e perfil lipídico (LDL-c).

**Conclusão:**

O tratamento com rosuvastatina não demonstrou diferença na redução do risco MACE em comparação com atorvastatina. Entretanto, a rosuvastatina pode ser mais eficaz do que a atorvastatina na redução dos níveis de LDL-c e não-HDL-c, além de promover aumento significativo no HDL-c. A rosuvastatina também demonstrou um perfil de segurança aceitável, com baixa incidência de eventos adversos graves. Assim, esta tecnologia pode ser uma opção terapêutica para esta população na redução dos níveis de LDL-c e aumento do HDL-c.

